# Artificial Intelligence in Contemporary Dentistry: Applications Across Dental Specialties and Future Challenges

**DOI:** 10.7759/cureus.117031

**Published:** 2026-09-27

**Authors:** Sumit Bhatt, Prantika Mandal, Shweta V Sagare, Sanjay J Gawali, Kunal Nischal, Sai C Das, Pratik Surana

**Affiliations:** 1 Department of Oral and Maxillofacial Surgery, Rajasthan Dental College and Hospital, Nirwan University, Jaipur, IND; 2 Department of Conservative Dentistry and Endodontics, North Bengal Dental College and Hospital, Sushrutanagar, IND; 3 Department of Conservative Dentistry and Endodontics, Bharati Vidyapeeth (Deemed to be University) Dental College and Hospital, Sangli, IND; 4 Department of Periodontology, Guru Gobind Singh College of Dental Sciences and Research Centre, Burhanpur, IND; 5 Department of Prosthodontics and Crown and Bridge, SGT Dental College and Hospital, Gurgaon, IND; 6 Department of Community Medicine, Kalinga Institute of Medical Sciences, KIIT University, Bhubaneswar, IND; 7 Department of Pedodontics and Preventive Dentistry, Maitri College of Dentistry and Research Centre, Durg, IND

**Keywords:** artificial intelligence, computer vision, deep learning, digital dentistry, machine learning

## Abstract

Modern artificial intelligence (AI) is increasingly shaping contemporary dental practice through efficient diagnosis, improved treatment planning, and patient personalization across various settings. By leveraging innovations in computer vision, deep learning, and multimodal pattern recognition, computational models are being applied across the major fields of dentistry today. These AI-based approaches are used for automated lesion detection in diagnostic imaging, precision alignment in orthodontics, root canal mapping in endodontics, and robotic assistance in surgical procedures. Despite the substantial improvements in diagnostic accuracy, objectivity, and efficiency, there are several major challenges with implementing these technologies into clinical practice. These challenges include algorithm bias, data privacy concerns, the absence of regulatory guidelines for the development of such tools, and cost. This review aims to provide an overview of the applications of artificial intelligence in the different fields of dentistry, to highlight the current challenges and directions for future research and development, and to discuss the potential of generative language models, real-time augmented reality (AR), and the prediction of dental healthcare outcomes.

## Introduction and background

During the last three decades, there has been an essential structural paradigm shift in dental medicine, one that has changed the nature of the diagnostic process from being primarily visual-tactile to completely digitalized. The broad implementation of intraoral scanning technology, digital panoramic imaging, high-resolution cone beam CT (CBCT) imaging, and computer-aided design/computer-aided manufacturing (CAD/CAM) systems has provided a firm basis for this kind of practice [1]. Nonetheless, with the exponential increase in diagnostic data, doctors have faced the problem of interpreting complex, multi-dimensional diagnostic files in the short amount of time available during consultations [2].

The combination of high-speed parallel processing, cloud computing, and vast annotated repositories of diagnostic data has brought artificial intelligence (AI) out of theoretical studies into practical use [3]. Rather than being limited to serving merely as a means of storing clinical information, current software tools analyze clinical data through neural networks. Through this process, clinical data is analyzed computationally to assist in objective diagnosis, minimize fatigue-related mistakes, standardize metrics, and improve treatment plans [4].

This narrative review presents an expanded evaluation of the contemporary landscape of AI integration across the primary dental specialties [5]. The key goal is to present a structured review of basic computer architecture, summarize recent clinical applications across various specialties, discuss the ethical, technical, and regulatory hurdles that hinder the technology's regular use, and examine technologies that will shape the future delivery of oral health care [6].

Although several recent reviews have addressed the applications of AI in dentistry, most have focused on isolated technologies, particular specialties, or general aspects of AI applications [1,2,5,7]. This narrative review aims to provide an updated and comprehensive overview of the use of AI across different dental specialties. It focuses on recent advances in areas of interest, including generative AI, augmented reality (AR), and predictive modeling for oral health care. Moreover, it also aims to highlight the associated challenges, ethical concerns, and limitations that may affect the implementation of these technologies in mainstream dental practice.

## Review

Literature search

A literature search was conducted to identify relevant publications that addressed the applications, developments, limitations, ethical considerations, and future perspectives of AI in dentistry. The search was carried out using PubMed/MEDLINE, Scopus, Web of Science, Embase, and Google Scholar. The literature search spanned publications from January 2015 to September 2026, with the final search conducted in September 2026.

The search strategy incorporated three major concepts: AI-related terminology, dentistry-related terminology, and specific clinical or technological applications. The main AI-related search terms included "artificial intelligence," "machine learning," "deep learning," "generative artificial intelligence," "generative AI," "neural networks," "large language models," and "predictive modeling." These terms were combined with dentistry-related terms, such as "dentistry," "dental," "oral health," "oral and maxillofacial radiology," "periodontology," "prosthodontics," "orthodontics," "endodontics," "oral and maxillofacial surgery," "pediatric dentistry," and "restorative dentistry." Further application-specific terms included "diagnosis," "dental imaging," "cone-beam computed tomography," "CAD/CAM," "treatment planning," "digital dentistry," "clinical decision support," "augmented reality," "virtual reality," "ethics," and "data privacy." Boolean operators (AND/OR) were used to combine these search concepts where appropriate.

The retrieved literature was initially screened by title and abstract for relevance to the objectives and scope of the narrative review. Potentially relevant articles were then assessed in full text. Publications were included if they provided relevant information on the development, clinical applications, diagnostic or therapeutic uses, technological advances, ethical and regulatory considerations, limitations, or future perspectives of AI in dentistry. Articles unrelated to the scope of the review or that did not contribute relevant information were excluded. The reference lists of relevant articles, particularly recent narrative and systematic reviews, were also manually screened to identify additional relevant publications. The final selection was based on relevance to the review's objectives, scientific contribution, and applicability to contemporary dental practice.

Fundamentals of AI in dental medicine

Core Computational Concepts and Subfields

An accurate definition of the integration of AI in oral health care depends on a clear differentiation among the basic subdisciplines and computational structures of AI [7]. AI is the general term within the field of computer science that involves building a computational structure capable of accomplishing tasks that usually require human intellect, including reasoning, problem solving, and visual perception [8]. Machine learning is a particular subdiscipline of AI that involves algorithms that identify underlying patterns in training data sets to produce predictions or classifications without hard-coded rules [9].

AI applications in dentistry can employ various approaches, including machine learning and deep learning, and these approaches can be applied to computer vision and natural language processing for analyzing dental data and information. Researchers have noted that deep learning is a particular type of machine learning that involves algorithms using many layers of processing, structured in a way inspired by the working principles of neurons in the human brain. Deep learning can thus be implemented using convolutional neural networks required for processing and analyzing two-dimensional images and three-dimensional volumes, taking into account all pixels or voxels [10].

Dental Data Modalities and Computational Processing

The efficiency of a dental AI system relies on the data input modality and its pre-processing procedure. There are several 2D radiography modalities, such as bitewing, periapical, and panoramic radiography, that use dense spatial pixel matrices to solve the problem of object detection, such as finding proximal carious lesions, periapical radiolucency, and marginal bone loss [11]. 3D Cone-Beam Computed Tomography offers volumetric voxel matrices and allows deep learning to be used to solve problems in volumetric segmentation, tracing internal canal morphologies, determining the inferior alveolar nerve canal, and evaluating alveolar bone density before dental implant placement [12].

In addition to radiographs, there is optical surface data obtained from an intraoral scanner, which provides dense three-dimensional meshes in stereolithography or wave-front object file formats [13]. A deep learning network analyzes these geometric meshes to assess the parameters of crown preparation, check the integrity of margin location, measure the dimensions of the arches, and track tooth wear progress. Standardized 2D clinical photographs, which provide high-resolution color data, help computer vision models to automatically classify soft tissue lesions, assess facial symmetry, and select intraoral shades [14].

Specialty-specific applications

Radiology and Diagnostics

Oral and Maxillofacial Radiology represents the first major area of dentistry in which deep learning is being applied. Convolutional Neural Networks trained on large databases of radiographs show a high level of sensitivity and specificity in diagnosing interproximal carious lesions, marginal gaps, and recurrent caries around restorations [15]. In addition, these tools can quantitatively evaluate periapical radiolucencies, classify periapical inflammatory lesions, and assess changes in bone density, among other uses. This could potentially reduce the impact of clinician-related fatigue and diagnostic variability, though more research is needed to confirm their efficacy and effectiveness in clinical settings [16].

Restorative Dentistry and Prosthodontics

Restorative dentistry and prosthodontics can benefit from incorporating AI algorithms into CAD/CAM software systems. Deep learning algorithms are capable of automatically recognizing finish lines, assessing occlusal clearance, and creating customized geometrical shapes of crowns corresponding to the morphology of the adjacent teeth [17]. Furthermore, machine learning algorithms for shade matching analyze digital photographs while accounting for the effects of environmental light, soft tissue reflection, and tooth translucency. Beyond the previously mentioned restorations, computer-aided design and computer-aided manufacturing are being used for removable partial dentures, complete dentures, implants, and maxillofacial prostheses. Occlusion and tooth wear analysis could also benefit from an AI-driven digital approach. Nevertheless, it is essential to assess the precision, fit, and performance of these AI-designed solutions against standard or manually designed alternatives [18].

Orthodontics

Orthodontics utilizes computer vision models extensively for cephalometric analysis and patient progress tracking. Machine learning programs can recognize hard and soft tissue landmarks from lateral cephalometric radiographs in seconds, compared with several minutes of manual work, while increasing the reproducibility of measurements. Treatment with clear aligners requires machine learning tools that analyze a series of intraoral scans to track the complicated dynamics of teeth movement, calculate biomechanical resistance, and detect deviations from the clinical path via remote photos [19].

Endodontics

Applying high-resolution deep learning models to CBCT volumes in endodontics improves pre-surgical anatomy assessment and diagnostic accuracy. AI is used to segment root canal systems and detect complex anatomical features such as C-shaped canals, lateral accessory canals, and extra root canals (for example, the mesiobuccal-2 canal in maxillary molars). In addition, these systems help detect small vertical root fractures, measure root curvature angles, calculate working lengths, and assess bone remineralization after endodontic treatment [20].

Periodontics

The diagnosis and assessment of periodontal conditions increasingly rely on deep learning methods to determine the severity and progression of disease. Using bitewing and panoramic radiographs, it is possible to assess interproximal alveolar bone levels relative to the cementoenamel junction and root apex, and estimate the extent of radiographic bone loss. These measures might be used in periodontal evaluation, though, according to the 2017 AAP/EFP classification, definitive periodontal staging requires consideration of clinical attachment loss, probing depth, radiographic bone loss, tooth loss due to periodontitis, and other relevant factors of disease complexity beyond radiographic bone loss. Deep learning tools applied to intraoral scans and images can also detect supragingival and subgingival calculus, marginal gingival inflammation, and gingival recession [21].

Oral and Maxillofacial Surgery

AI is used extensively in oral and maxillofacial surgery for pre-surgical risk estimation and guiding procedures. A deep learning algorithm is used to map the exact three-dimensional course of the inferior alveolar nerve canal in relation to impacted third molar roots, lowering the risk of nerve injury. During reconstruction and oncological procedures, AI helps demarcate surgical resection limits, osteotomy sites, and patient-specific surgical guides and titanium implants for maxillofacial reconstruction [22].

Pediatric Dentistry

AI is a promising tool in pediatric dentistry for the early detection of caries, radiographic diagnostic analysis, assessment of craniofacial growth, treatment planning, and prediction of caries risk. Algorithms for image processing can improve diagnostic accuracy for primary and secondary caries and standardize radiographic interpretations. Additionally, AI can integrate variables such as dental, demographic, dietary, and behavioral information to predict caries risk in individuals. In orthodontics and dental anthropology, AI can automatically identify anatomical landmarks, analyze growth trends, and assess craniofacial morphology.

Notably, caries diagnosis and management in pediatrics present unique challenges absent in caries detection and management in adults. Because primary teeth, mixed dentition, developing permanent teeth, and even anatomical features of the jaws and skulls change drastically with age, the performance of a single algorithm may vary considerably. Therefore, a critical direction for future research is developing algorithms applicable across different age groups and validating their performance in different populations. Other research directions should focus on analyzing the pediatric population at risk for caries, determining caries progression at an early stage, analyzing behavior and anxiety in young patients, and using multimodal AI diagnostics. Finally, there is a need for prospective validation studies to evaluate the clinical benefits of various AI applications in the diagnosis and management of pediatric patients [5,7].

AI in Oral Pathology and Oral Cancer Screening

AI-based image analysis in oral pathology and oral cancer screening has attracted growing attention in recent years. Machine learning and deep learning algorithms may assist in detecting and characterizing potentially malignant and malignant oral lesions from clinical photographs and histopathological or radiographic imaging, facilitating early diagnosis, lesion characterization, and diagnostic decision-making. Nevertheless, such applications require clinical validation across a variety of patient cohorts and imaging conditions to optimize diagnostic accuracy and reliability in practice [5].

AI in Implantology

AI applications are increasingly being explored in implant dentistry for treatment planning, implant positioning, anatomical assessment, and prediction of treatment-related outcomes. AI-driven analysis of three-dimensional imaging data, including CBCT, can support identifying relevant anatomical landmarks and aid individualized implant planning, increasing efficiency and accuracy. However, clinical validation and standardization of these approaches remain essential for successful clinical integration.

AI in Temporomandibular Disorders

AI applications for temporomandibular disorders (TMDs) focus primarily on image analysis, pattern recognition, diagnosis, and treatment outcome prediction. Applying machine learning algorithms to TMD research has demonstrated potential for identifying relevant clinical and imaging findings associated with TMDs, supporting clinical assessment and diagnosis. However, the complexity of TMDs and variations in diagnostic criteria present challenges to consistent and reliable AI-based diagnosis and management [20].

Current challenges and limitations

Data Privacy, Security, and Informed Consent

Incorporating AI into dental healthcare presents significant data privacy and cybersecurity issues. Three-dimensional volumes, such as CBCT images or facial surface scans, may contain sensitive anatomical information and thus require appropriate security measures for storage, processing, and dissemination, in compliance with HIPAA and GDPR. Proper data storage and encryption, along with proper data de-identification protocols, are needed to ensure patient data protection, while clinical practices need to develop an informed consent process for data use [5,23].

Algorithmic Bias, Data Quality, and Generalizability

A major limitation of AI applications in dentistry is the potential for various forms of algorithmic bias. Selection bias may occur when training datasets fail to adequately represent the target patient population and can be assessed by comparing the demographic and clinical characteristics of the development dataset with those of the intended clinical population. Dataset shift may arise when differences in imaging devices, acquisition protocols, clinical settings, or patient populations cause model performance to vary between training and deployment data; this can be evaluated through external validation across independent datasets and settings. Label bias can occur when diagnostic labels or reference standards are inconsistent or influenced by subjective interpretation, and can be assessed through standardized reference criteria and inter-rater agreement. Subgroup fairness relates to differences in model performance across demographic or clinically relevant subgroups and can be evaluated by comparing measures such as sensitivity, specificity, and other performance metrics across these groups [24].

Regulatory Frameworks, Standardization, and Medicolegal Liability

Legal uncertainty still exists around regulating AI-enabled medical devices, creating legal dilemmas in cases of misdiagnosis, over- or under-treatment. When an autonomous or semi-autonomous AI-based diagnostic system produces erroneous recommendations, responsibility depends on the jurisdiction, the classification of devices, the intended use, and the specific case. Liability may fall on doctors, hospitals, manufacturers, or developers depending on the circumstances. Therefore, explainable AI is necessary to explain algorithms' decisions [25].

Implementation Barriers: Cost, Integration, and Clinician Training

Translating AI software from research into practical clinical application faces numerous monetary and logistical challenges. These include licensing costs for software, hardware upgrades, and the integration of third-party platforms with existing practice management software. Further, the absence of standard interface design and the need to train clinical staff on the software result in time limitations for high-throughput clinics [26].

Future perspectives and emerging horizons

Generative AI and Large Language Models in Dental Practice

The rapid development of generative AI and large language models offers opportunities to optimize non-clinical administration and patient communication. However, generative AI use can lead to the fabrication of false or inaccurate information, commonly referred to as "hallucination." Before using any information generated by AI, the data must be verified by qualified dental professionals. A fine-tuned language model can automate collecting detailed medical history, structure unstructured clinical notes for the electronic health record system, and provide clear, personalized explanations of treatment to patients [27].

Real-Time Augmented Reality and Robotic-Assisted Surgery

Integrating computer vision with AR and surgical robotics is changing how guidance is achieved in dental surgeries [28]. Real-time three-dimensional imaging overlaid on an AR headset allows surgeons to view anatomical structures not normally visible, including nerves, maxillary sinus borders, and root tips, directly within their field of view during the procedure. In addition, AI-powered haptic robots provide consistent and highly accurate guidance for procedures such as implants, endodontics, and orthognathic surgery [29].

Personalized Predictive Oral Healthcare

Future developments in dental AI will likely evolve from a reactive model to a more proactive, personalized, and predictive approach to dental care. This is because machine learning techniques can predict disease risks by utilizing data from longitudinal images, intraoral scans, medical histories, saliva-based diagnostics, and genetics. Such an approach will facilitate the creation of preventive treatment programs.

Discussion

The growing body of evidence indicates that AI is no longer confined to experimental image-recognition systems and has expanded into broader applications in diagnosis, treatment planning, education, research, and clinical workflow. However, this progress must be interpreted cautiously, since reported technical performance does not necessarily translate into better clinical outcomes. Recent evidence syntheses have demonstrated that AI generally performs well in diagnosis across dental applications, but considerable heterogeneity exists between studies and clinical tasks. This heterogeneity suggests that pooled measures of accuracy should not be taken as evidence of uniform clinical effectiveness across dental settings.

Translating Diagnostic Accuracy into Clinical Effectiveness

An important distinction in dental AI research lies between algorithmic accuracy and clinical effectiveness. Many studies evaluate AI using retrospective radiographic or photographic datasets, but far fewer studies evaluate AI's performance in a real clinical workflow [30]. A technically accurate algorithm may have limited clinical utility due to increased interpretation time, excessive false positive findings, or a lack of impact on clinical decision-making. A recent review of AI use in dentistry found that most studies were retrospective or validation studies, with few prospective evaluations in real-world settings. This underscores the need to shift research priorities away from demonstrating that an algorithm can perform a task toward whether its use actually improves patient care.

Future studies should therefore incorporate clinically meaningful endpoints, such as changes in diagnostic decisions, treatment planning, referral patterns, procedural efficiency, patient outcomes, and cost-effectiveness. Single-center retrospective studies may carry greater limitations in bias, reproducibility, and generalizability than multicenter AI studies. Studies should assess AI within real clinical settings, considering differences in imaging devices, operators, patient populations, disease prevalence, and workflow complexity.

Collaboration, Not Replacement, of Humans and AI

Available evidence supports a model in which AI acts as a clinical decision-support technology rather than a replacement for dental professionals. The diagnosis of dental disease is often made by combining radiographic findings with the results of a clinical examination, the patient's symptoms, medical history, previous treatments, patient preferences, and contextual factors unavailable in computational datasets. Therefore, even the most sophisticated models may provide only partial evaluations when used alone.

Human-AI collaboration may be particularly useful when AI systems serve as a second reader, abnormality detector, measurement assistant, or risk-stratification tool [31]. These applications could reduce repetitive cognitive tasks and allow clinicians to retain responsibility for interpretation and final decision-making. However, for collaboration to be effective, clinicians must understand the capabilities and limitations of the underlying technology. Similar findings appear in recent literature, emphasizing that future dental AI should be integrated into clinical workflows as a transparent, validated decision-support tool rather than a stand-alone diagnostic authority.

Automation Bias and the Need for Critical Clinical Supervision

As AI use grows, there is a risk of "automation bias," in which clinicians may accept computer recommendations too readily without independently checking them. This problem becomes particularly significant when AI systems make highly confident predictions despite uncertainty or incomplete input data. Relying too heavily on automated outputs may introduce a new source of diagnostic error, rather than simply eliminating existing variability.

Thus, AI interfaces should communicate uncertainty and provide clinically interpretable information wherever possible. Dental professionals should be encouraged to challenge AI recommendations when they conflict with clinical findings. Training must therefore focus not only on the technical use of AI platforms but also on the critical appraisal of their outputs, recognizing inappropriate recommendations, and identifying situations where human judgment should take precedence.

External Validation, Diversity of Datasets, and Robustness of Models

The generalizability of dental AI remains highly dependent on the diversity and quality of training and validation datasets. Factors such as patient characteristics, disease prevalence, imaging protocols, sensor technologies, image quality, and institutional practices may affect model performance. Thus, good performance on a development set should not be assumed to imply good performance on another population.

Recent literature has emphasized the importance of external validation and standardized evaluation. A recent evidence synthesis has highlighted the need for methodological standardization, reporting considerable heterogeneity in sensitivity, specificity, area under the curve, and accuracy across dental AI studies [32]. Multicenter datasets encompassing geographically, demographically, and technically diverse populations may increase model robustness and reduce the risk of overfitting to a particular clinical environment.

Artificial Intelligence in Dental Education and Continuing Education

Beyond its immediate clinical applications, AI may play a significant role in educating and training dental practitioners. AI-enabled systems can support personalized instruction, automated feedback, training in radiographic interpretation, simulation-based education, and the generation of educational content. However, current evidence suggests AI is less capable of assessing complex competencies such as clinical judgment, manual dexterity, communication, and contextual decision-making. Dental curricula may need to expand training in AI literacy, including how to interpret model limitations, data bias, uncertainty, privacy, responsible use, and the validation of AI-generated information.

Integration and Interoperability With Dental Information Systems

Interoperability with existing digital dental infrastructure is another key factor for successful AI implementation. AI tools that are not integrated with electronic dental records, imaging platforms, intraoral scanners, or practice-management systems can add complexity to workflows. Smooth integration would allow access to relevant patient information without repetitive data entry, and would enable longitudinal analysis of radiographs, clinical photographs, treatment histories, and other digital records.

Future development should therefore focus on interoperable systems that can exchange standardized clinical information across platforms. Such integration may also enable longitudinal monitoring, in which AI models can be used to detect changes in oral health status, rather than relying on discrete clinical encounters [33,34].

Economic and Equity Issues

The clinical value of dental AI should also be evaluated from a health-economic and equity perspective. Advanced AI systems may require special hardware, cloud infrastructure, software subscriptions, technical support, and ongoing model updates. These requirements may be prohibitive for adoption in smaller practices and resource-limited healthcare systems. Technological advancement does not, therefore, automatically guarantee equal access to AI-assisted oral healthcare.

A key future direction is the development of scalable, affordable AI systems compatible with different levels of healthcare infrastructure. AI could provide underserved populations with remote image interpretation, initial risk assessment, and prioritization of patients needing specialist evaluation. However, such applications require appropriate validation in the populations for which they are intended, rather than assuming that models developed in highly resourced settings will perform equivalently elsewhere.

Monitoring During Clinical Implementation

Initial regulatory or clinical assessment should not be considered as permanent validation for AI systems. Model performance may change over time due to changes in patient populations, disease patterns, imaging devices, software environments, and clinical workflows. Continuous surveillance after deployment could therefore be an important component of AI governance.

Future AI frameworks in dentistry should incorporate ways to detect performance decline, log clinically serious errors, monitor for unexpected results, and update models as needed. This would shift AI evaluation from a one-off validation process toward a continuous lifecycle model of quality assurance. This is especially important for adaptive or regularly-updated systems, where model behavior may shift due to software modifications or new training data.

Directions for Future Research

Together, the evidence suggests that the next phase of dental AI research should move beyond proof-of-concept development toward clinically grounded implementation science. Priority areas include prospective multicenter validation, standardized reporting of AI performance, evaluation of human-AI interaction, health-economic assessment, longitudinal outcome studies, and systematic post-deployment monitoring. It will also require far greater collaboration among dentists, computer scientists, statisticians, ethicists, regulatory specialists, and patients to ensure that AI development addresses clinically meaningful problems.

## Conclusions

The application of AI is increasingly shaping modern-day dentistry into a more data-informed clinical practice. Although existing deep learning systems have shown promising performance in supporting clinical decision-making in radiology, restorative dentistry, orthodontics, endodontics, periodontics, and oral surgery, these systems serve as clinical decision-making supports intended to support, not replace, practitioners' decisions. Addressing challenges in algorithm generalizability, data confidentiality, legal issues, and the expenses of implementation is key to the safe and effective integration of AI.
